# Association of patellofemoral morphology and alignment with the radiographic severity of patellofemoral osteoarthritis

**DOI:** 10.1186/s13018-021-02681-2

**Published:** 2021-09-04

**Authors:** Yike Dai, Heyong Yin, Chongyang Xu, Hongrui Zhang, Ai Guo, Naicheng Diao

**Affiliations:** Department of Orthopedic, Beijing Friendship Hospital, Capital Medical University, No. 95 Yong’an Road, Xicheng, Beijing, 100050 People’s Republic of China

**Keywords:** Patellofemoral osteoarthritis, Sulcus angle, Congruence angle, Patellar tilt, Patella height

## Abstract

**Background:**

Risk factors for the severity of patellofemoral osteoarthritis (PFOA) are poorly understood. This research aims to evaluate the association between patellofemoral joint (PFJ) morphology and alignment with the radiographic severity of PFOA.

**Methods:**

A retrospective analysis of CT scan and lateral radiograph data were acquired in patients with PFOA. The radiographic grade of PFOA and tibiofemoral osteoarthritis (TFOA), lateral and medial trochlear inclination angle, sulcus angle, and the Wiberg classification of patella morphology, the congruence angle, patellar tilt angle, and lateral patellar angles, and tibial tubercle trochlear groove distance (TT-TG) and patella height (i.e., Caton-Deschamps index) were assessed using CT scans and sagittal radiographs of the knee. All the PFJ morphology and alignment data were divided into quarters, and the relationships between each of these measures and the severity of PFOA were investigated.

**Results:**

By studying 150 patients with PFOA, we found a U-shaped relationship between the Caton-Deschamps index and the severity of PFOA (*P* < 0.001). A lower value of sulcus angle and lateral patellar angle, a higher value of congruence angle, and type III patella were associated with more severity of lateral PFOA. Compared with the highest quarter of each measure, the adjusted odds ratios (OR) of the severity of PFOA in the lowest quarter of sulcus angle, lateral patellar angle, and congruence angle; and type I patella was 8.80 (*p* = 0.043), 16.51 (*P* < 0.001), 0.04 (*P* < 0.001), and 0.18 (*p* = 0.048) respectively.

**Conclusions:**

Extreme value of patella height, a higher value of lateral patellar displacement and lateral patellar tilt, lower value of sulcus angle, and type III patella were associated with more severity of PFOA.

## Background

Patellofemoral joint osteoarthritis (PFOA) is a type of knee OA, which is one of the most commonly affected compartments. Approximately, 50% of patients with knee joint osteoarthritis (KOA) do have some degree of PFOA. The prevalence of PFOA appears to be higher in females (age ≥ 50 years) (41%) than in males (age ≥ 50 years) (23%) [1]. The most common radiographic findings in patients with knee symptoms were combined tibiofemoral and patellofemoral joint (PFJ) lesions (40%), followed by isolated PFOA (24%), isolated tibiofemoral joint osteoarthritis (TFOA) only 4%, and the remaining 32% had normal radiographic findings [2]. Previously, studies have shown that PFOA most frequently involves the lateral compartment of PFJ [3, 4]. Some studies suggested the risk factors associated with the disease are different for PFOA and TFOA [5, 6]. Different from radiographic TFOA, the risk factors of the radiographic severity of PFOA have not been as thoroughly studied.

Abnormal PFJ alignment and morphology are hypothesized to alter the mechanics of the PFJ, which can in turn lead to PFOA. PFJ malalignment typically manifests as lateral patellar tilt, lateral displacement, or a combination of the two, which is a deviation of the patella relative to trochlea that may lead to abnormal stress transmitted through the PFJ [7]. A previous study identified the association between PFOA and both abnormal trochlear sulcus morphology and tibiofemoral alignment [8]. However, there is little evidence that patella height, lateral patellar displacement, and patellar tilt are associated with PFOA. A previous study indicated that the vertical position of the patella is associated with patellofemoral malalignment and reduced PFJ contact area in patella Alta [9]. We speculate that the increasing of patella height, sulcus angle (i.e., a flatter trochlear), lateral patellar displacement, and lateral patellar tilt will lead to patella instability and easily cause PFOA. Besides, according to Wiberg’s investigations, the patella shape is defined into three types [10]. However, there are few studies about the relationship between patella morphology with the severity of PFOA.

The purpose of this study was to determine the association of PFJ alignment (patella height, lateral patellar displacement, patellar tilt, and lateral patellar angle, TT-TG) and PFJ morphologic features (sulcus angle, medial trochlear inclination angle, lateral trochlear inclination angle, trochlear angle, and patella shape) with the severity of PFOA.

## Methods

This study has been approved by the Ethics Committee of our hospital. CT scans and lateral radiograph data of 165 patients with PFOA were analyzed retrospectively between June 2019 and June 2020 at our hospital. All the cases in this study were hospitalized for total knee arthroplasty or arthroscopic surgery. CT scans of the knee joints were performed in the supine position with the knee in extension and lateral radiographs were taken in full weight-bearing holding knees flexed 30° and feet externally rotated 10°. We staged the severity of PFOA based on Merchant et al. [11]. CT scans were performed to define PFOA due to the PFOA criteria for MRI definition are not well established, and MRI features of PFOA such as PFJ marrow edema and minor cartilage defects are common even in young asymptomatic knees [12]. To be included, participants in this cohort had to have a diagnosis of PFOA confirmed by preoperative CT scan and intraoperative observation, as well as all the cases should have preoperative CT and lateral radiographs. The exclusion criteria of this study include (1) traumatic PFOA, (2) patients without CT or lateral radiographs, and (3) previous surgery to the lower extremity. Of the 165 knees analyzed in this study, 15 were excluded due to the inability to identify skeletal landmarks. This occurred because of severe osteoarthritis with severe osteophytes. This left 150 knees (150 patients) eligible for the study. The demographic data of the 150 patients are shown in Table 1.

**Table 1:** Patients demographics (*n* = 150 knees, 150 patients)

Variable (mean ± SD)	***N*** (%)
Age, years	60.7 ± 9.5
Gender, F	108 (72%)
Side, right	78 (52%)
Sulcus angle	150.6° ± 9.3°
Lateral trochlear inclination	16.7° ± 5.2°
Medial trochlear inclination	12.5° ± 6.7°
Wiberg classification	
Type I	54 (36%)
Type II	75 (50%)
Type III	21 (14%)
Congruence angle	26.4° ± 21.7°
Patella tilt	15.4° ± 8.3°
Lateral patellar angle	1.6° ± 6.9°
Caton index	0.9 ± 0.1
TT-TG	14.9 mm ± 4.8 mm

*TT-TG* tibial tubercle–trochlear groove distance

### Assessment of radiographic OA

Using the CT scan, each knee was evaluated for joint space stenoses of PFJ on a 1-4 stage based on Merchant et al. [11]. Stage 1 is mild, with the joint space of at least 3 mm; stage 2 is moderate, with the joint space less than 3 mm, but no bone contact; stage 3 is severe, with partial bony contact less than 1/4 of the joint surface; and stage 4 is very severe, and the surface of the joint bone is completely touching each other. In this study, we combined stage 3 and stage 4 PFOA into stage 3. We only assessed the severity of the lateral PFOA, not the medial PFOA, because we did not find a case with isolated medial PFOA through further intraoperative observation. Besides, the severity of TFOA was also evaluated to exclude the interference to PFOA. The radiographic TFOA was evaluated by using the Kellgren and Lawrence grade [13].

### Assessment of patellofemoral joint morphology and patellofemoral alignment

**Fig. 1:**
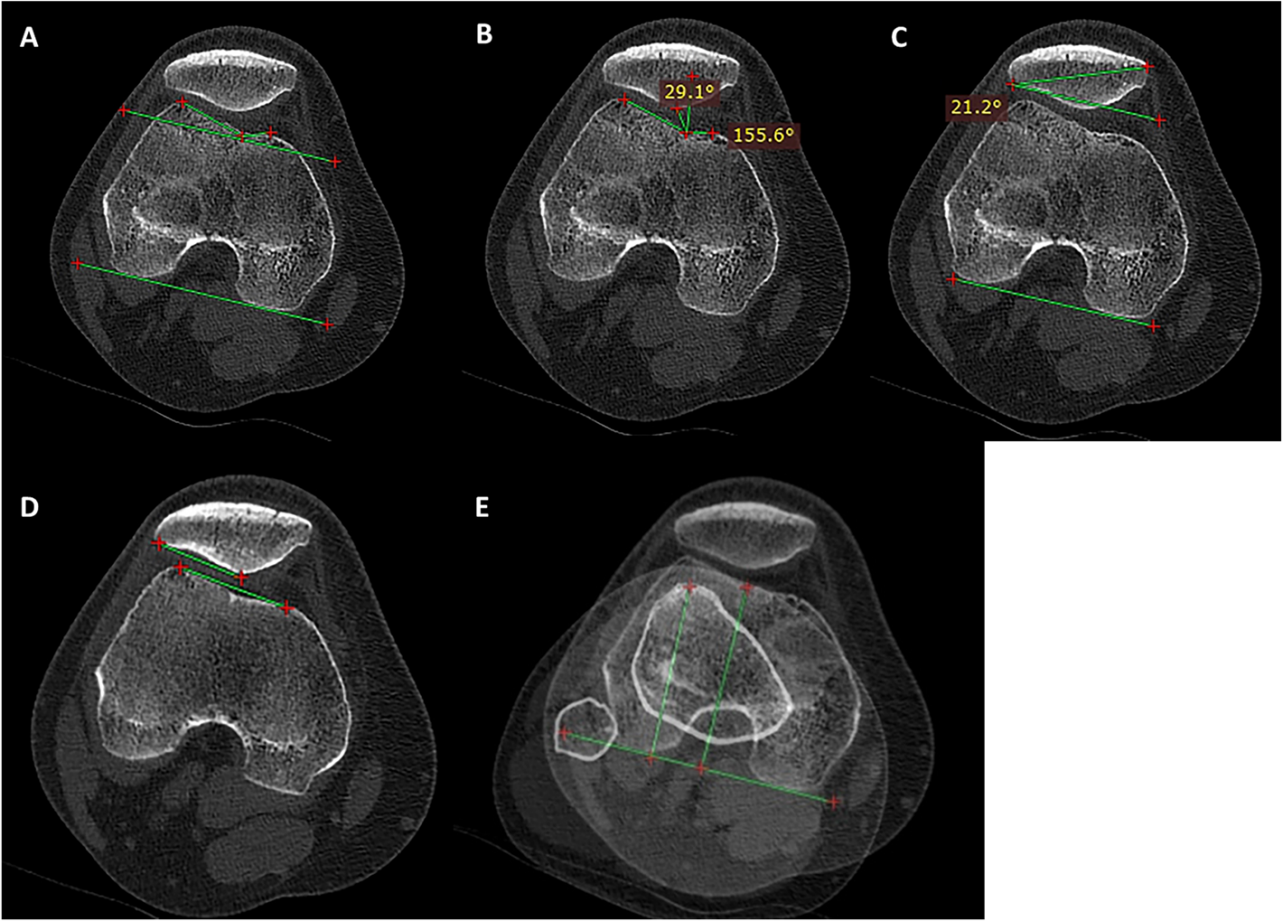
(**A**) Measurement of PFJ morphology. (**B**) Measurement of congruence angle. (**C**) Measurement of patella tilt. (**D**) Measurement of lateral patellar angle. (**E**) Measurement of TT-TG

We measured three kinds of morphologic characteristics of the trochlea: the lateral and medial trochlear inclination angle, and sulcus angle (Fig. 1A). The CT image of trochlear morphology was analyzed in the section of the “Roma Dome.” The morphology of the patella is classified by Wiberg classification according to CT images [10]. In type I, the medial and lateral facets are symmetrical, and of equal size. In type II, the medial facet is smaller and flatter than the lateral facet. In type III, the medial facet is convex, more vertical, and significantly smaller.

**Fig. 2:**
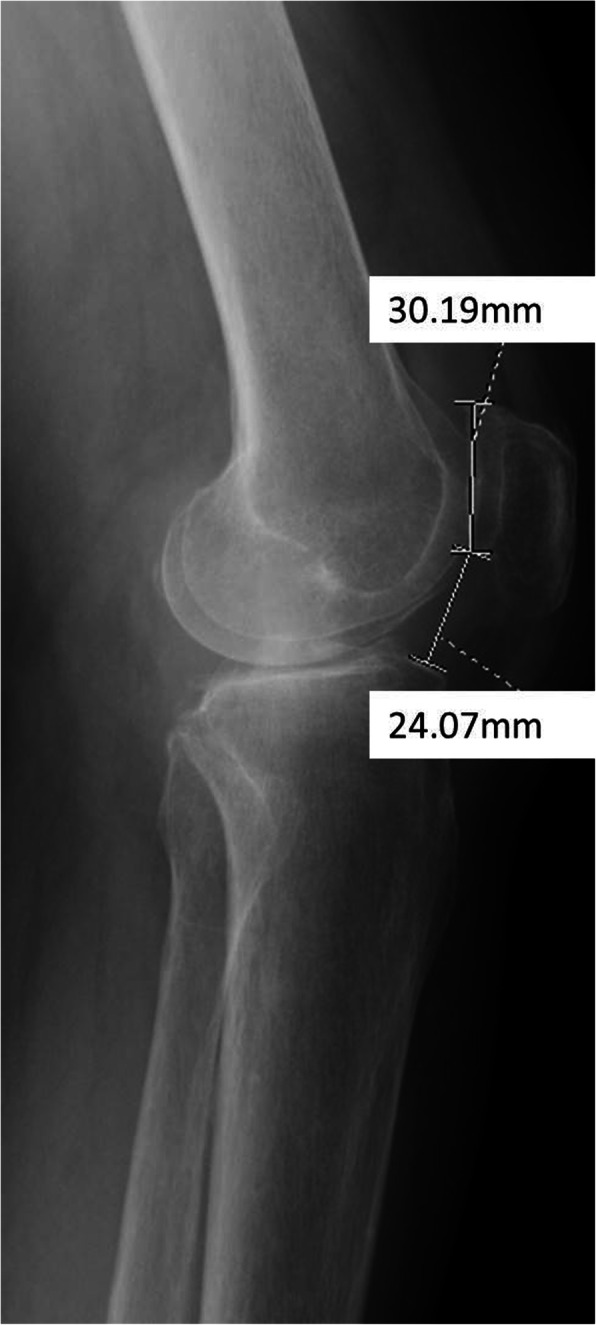
Measurement of patella height (Caton-Deschamps index)

We measured five different aspects of the patellofemoral alignment: The congruence angle (Fig. 1B), patellar tilt (Fig. 1C), and lateral patellar angles (Fig. 1D); patella height (i.e., Caton-Deschamps index) (Fig. 2) and TT-TG (Fig. 1E). Morphologic features of the trochlea, lateral displacement (the congruence angle), patellar tilt, and lateral patellar angles were measured on a CT scan [14]. Patella height (Caton index) [15] was measured on the full weight-bearing lateral radiographs. TT-TG was measured by two scanning slices, and the method with a technique was described by Schoettle et al. [16]. Specific methods are depicted in Table 2.

**Table 2:** Measurement methods of the parameters

Parameters		Definition
**PFJ morphology**		
Sulcus angle	SA	This angle is defined by lines joining the highest points of the medial and lateral condyles and the lowest point of the intercondylar sulcus
Lateral trochlear inclination	LTI	The angle between the lateral facet line of trochlea and posterior condylar line
Medial trochlear inclination	MTI	The angle between the medial facet line of trochlea and posterior condylar line
Wiberg classification	Wiberg	Type I, the facets are symmetrical, concave, and of equal size Type II, the medial facet is smaller and flatter compared to the lateral facet Type III, the medial facet is convex, more vertical, and markedly smaller
**PFJ alignment**		
Congruence angle	CA	The line through the apex of the patella to a line bisecting the trochlea
Patella tilt	PT	The angle between the posterior condylar line and the maximal patella width line
Lateral patellar angle	LPA	The angle between the lateral facet line of patella and the line joining the highest points of the medial and lateral condyles
Caton-Deschamps index	CDI	The ratio between the length of the distance from the lower edge of the patellar articular surface to the anterior superior surface of the tibial plateau and the length of the patellar articular surface
Tibial tubercle–trochlear groove distance	TT-TG	The TT-TG distance is the distance from the deepest point of the trochlear groove to the highest point of the tibial tuberosity

*PFJ* patellofemoral joint

### Statistical analysis

All statistics were analyzed by the SPSS software (IBM, Armonk, NY, USA, v. 24.0). We randomly selected 30 knees to test the intra- and interobserver reliability. Two experienced surgeons using the same standard criteria independently measured the data twice with an interval of 4 weeks and then calculated the intra- and interclass correlation coefficient (ICC). After a normal distribution was ensured, the relationship between all the measures of patellofemoral morphology and alignment with the radiographic severity of lateral PFOA was explored by using conditional multivariable logistic regression (the grading III as the referent category). To avoid the effect of the degree of TFOA on the outcome, radiographic TFOA was adjusted in the multivariable regression model. A power analysis was undertaken. At least 138 cases were needed to reach an effect size of 0.15, a power of 0.95, and an alpha of 0.05 using G*Power v. 3.1 (G* Power, Dusseldorf, Germany). Statistical significance was set at *p* < 0.05.

## Results

At last, a total of 150 knees (150 patients) were included in this study, including 108 females (72%) and 42 males (28%), 78 (52%) right knees, and 72 (48%) left knees. The mean age was 60.7 years (SD = 9.5) (Table 1). The results characterizing the patellofemoral morphology and alignment are shown in Table 1. The ICC results ranged from 0.838 to 0.969, which indicated excellent agreement (Table 3).

**Table 3:** Results of radiological parameters of PFJ morphology and alignment and interclass correlation coefficient tests

Parameters	Mean (SD; range)	Interobserver ICC (95% CI)	Intraobserver ICC (95% CI)
Joint space narrowing	1.9 mm (1.1 mm; 0 mm to 4.47 mm)	0.956 (0.906 to 0.979)	0.962 (0.920 to 0.982)
**PFJ morphology**			
Sulcus angle	150.6°(9.3°; 124° to 173°)	0.878 (0.703 to 0.939)	0.906 (0.800 to 0.956)
Lateral trochlear inclination	16.7° (5.2°; 4° to 29°)	0.852(0.684 to 0.930)	0.948 (0.876 to 0.941)
Medial trochlear inclination	12.5° (6.7°; 1° to 36°)	0.845 (0.651 to 0.947)	0.939 (0.870 to 0.971)
Wiberg classification	_	0.865 (0.712 to 0.937)	0.869 (0.721 to 0.938)
**PFJ alignment**			
Congruence angle	26.4° (21.7°; -38 to 74)	0.860 (0.678 to 0.921)	0.964 (0.922 to 0.983)
Patella tilt	15.4° (8.3°; 0 to 43)	0.969 (0.934 to 0.985)	0.935 (0.863 to 970)
Lateral patellar angle	1.63° (6.9°; -28° to 16°)	0.915 (0.781 to 0.912)	0.913 (0.815 to 959)
Caton-Deschamps index	0.9 (0.1; 0.60 to 1.34)	0.838 (0.655 to 0.924)	0.914 (0.816 to 0.960)
TT-TG	14.9 (4.8; 0.0 to 28.9)	0.919 (0.827 to 0.962)	0.974 (0.945 to 0.988)

*PFJ* patellofemoral joint, *TT-TG* tibial tubercle–trochlear groove distance

The relationship between patellofemoral alignment and morphology with the radiographic severity of lateral PFOA (the grading 3 as the referent category) is shown in Table 4. The sulcus angle and lateral patellar angle were negatively correlated with the radiographic severity of lateral PFOA, while the congruence angle and patella morphology were positively correlated with the radiographic severity of lateral PFOA. More notably, a statistically U-shaped relationship was observed between the patellar height and severity of lateral PFOA, which indicated that the highest or lowest quarter of patella height has a higher incidence of more severe lateral PFOA than the middle two-quarters of the patellar height (*p* = 0.011).

**Table 4:** Relationship between patellofemoral alignment and morphology with the radiographic severity of lateral PFOA (the grading 3 of PFOA as the referent category)

Measures	Quarter	Range	***N*** of cases	OR	Adjusted OR
SA (degree)	1	124.0-142.0	44	7.66	8.80
	2	142.1-150.0	36	5.14	6.00
	3	150.1-157.3	38	1.57	1.37
	4	157.4-173.0	32	1.00	1.00
Wiberg classification	I	-	54	0.23	0.18
	II	-	75	0.48	0.39
	III	-	21	1.00	1.00
CA (degree)	1	−38.0-9.0	37	0.06	0.04
	2	9.1-18.5	43	0.25	0.22
	3	18.6-33.8	35	0.47	0.43
	4	33.9-58.0	35	1.00	1.00
CDI	1	0.60-0.78	35	1.75	1.21
	2	0.79-0.91	39	0.84	0.70
	3	0.91-1.03	36	0.72	0.52
	4	1.03-1.39	40	1.00	1.00
LPA (degree)	1	−15.0-1.0	38	17.55	16.51
	2	1.1-4.0	37	2.54	2.52
	3	4.1-7.0	33	2.12	2.08
	4	7.1-16.0	42	1.00	1.00

*SA* sulcus angle, *CA* congruence angle, *LPA* lateral patellar angle, *CDI* Caton-Deschamps index

When compared with the highest quarter of the sulcus angle and lateral patellar angle, the adjusted ORs of the radiographic grade 3 of lateral PFOA were 8.8 for the lowest quarter of the sulcus angle (*P* = 0.043) and 16.51 for the lowest quarter of the lateral patellar angle (*P* < 0.001), respectively. Similarly, when compared with the highest quarter of the congruence angle and type III patellar morphology, the adjusted ORs of the radiographic grade 3 of lateral PFOA were 0.04 for the lowest quarter of the congruence angle (*P* < 0.001) and 0.18 for the type I patellar morphology(*P* = 0.048), respectively (Table 4). Besides, when radiographic TFOA is adjusted, there is no change in the results (adjusted OR) substantially.

## Discussion

In this study, we evaluated various measures of PFJ morphology and alignment to determine which could predict the radiographic severity of lateral PFOA. The most important findings of the present study were as follows: (1) deeper trochlea (smaller sulcus angle), patellar lateral displacement (larger congruence angle), and patellar lateral tilt (smaller lateral patellar angle) are associated with more severe lateral PFOA; (2) type III patellar morphology is associated with more severe lateral PFOA; and (3) a statistically U-shaped relationship between the patellar height (Caton-Deschamps index) and severity of lateral PFOA.

Previous studies identified that most of the variability in alignment can be explained by the combination of morphology parameters, highlighting the complex interaction between patellofemoral joint morphology and alignment [17, 18]. These intricate interactions between morphology and alignment may result in pathologic alterations of the patellofemoral joint. In most patients, such a multifaceted etiology makes the identification of a single pathological factor for PFOA extremely challenging. Therefore, we evaluated PFJ morphology and alignment to determine which predicted the radiographic severity of lateral PFOA. All the cases in this study were hospitalized for total knee arthroplasty or arthroscopic surgery. Through further intraoperative observation, we did not find a case with isolated medial PFOA, possibly because the medial patellar instability is an objective iatrogenic condition [19]. Therefore, we just analyzed the influencing factors for the severity of lateral PFOA in all cases. In this study, we identified a statistically U-shaped relationship between the patellar height (Caton-Deschamps index) and severity of lateral PFOA, which suggested that extreme values of the patellar height have a higher incidence of more severe lateral PFOA. Several previous studies suggested the patella Alta as a possible risk factor for PFOA [20, 21], while some other studies suggested that patella Baja can produce increased patellofemoral contact pressures at early knee flexion, which can potentiate arthritis progression [22, 23]. We propose several explanations for this finding. On the one hand, a higher sagittal position of the patella would lead to a reduced PFJ contact area during knee extension. These abnormal mechanical mechanics of PFJ can lead to patellar instability, PFJ malalignment, and increased shear forces and PFJ contact pressure, which may be risk factors for PFOA [24, 25]. On the other hand, a lower patella may create excessive stress on the articular surface at knee flexion, which causes degeneration of chondrocytes and contributes to the formation of PFOA.

Furthermore, our study also suggested that a smaller sulcus angle (a narrow trochlea) increased the severity of lateral PFOA, but not flatter trochlea. This is different from our speculation that greater sulcus angle increased prevalent PFOA, which was identified in some previous studies [26, 27]. Ali et al. indicated that a larger sulcus angle was associated with severe cartilage defects [28]. Similarly, another study indicated an association between larger sulcus angle and PFJ cartilage injury [27]. However, our study identified a different view that narrow trochlea increased prevalent PFOA. We hypothesized that a narrow trochlea may produce a smaller contact area in PFJ, resulting in excessive stress on the PFJ surface. Meanwhile, shallower trochlear sulcus could increase the PFJ contact area and reduce the articular surface contact pressure, which produces a better distribution of the stress on PFJ.

The association of the patella morphology and PFOA has only been sparsely reported in the literature. In our patient population, the patella type, classified according to Wiberg, showed a significant prevalence of type II patella (74%), followed by type I patella (36%) and type III patella (14%). In this study, type III patellar morphology is associated with more severe lateral PFOA. A previous study showed that Wiberg patella type III, which presented a medial border dysplasia or a short patellar apex, is more often involved in patients with patellar dislocation [29], and patella lateral displacement is associated with more severe lateral PFOA [30]. On the other hand, Wiberg type III patella presented only a small amount of contact with the medial femoral condyle due to the low surface area of the medial facet, which increased the contact pressure of the lateral patella facet on the lateral femoral condyle during knee flexion.

Besides, we also identified that congruence angle was associated with the severity of lateral PFOA positively, while lateral patellar angle was associated with the severity of lateral PFOA negatively. These results are consistent with the results of previous studies that patella lateral displacement or patella tilt could reduce the contact area of the PFJ and increased contact stress on the PFJ surface [31, 32]. Excessive stress on the lateral articular surface of the PFJ could increase the risk of lateral PFOA. This may be why previous studies recommended applying a knee taping to force the patella medially and away from the overloaded lateral compartment as a treatment for PFOA [33, 34].

Several strengths of our study are remarkable. First, the CT scan of the knee was used to define the radiographic PFOA rather than the skyline view of knee radiographs. CT image facilitates the evaluation of radiographic PFOA and measurement morphology and alignment of PFJ. Second, it is worth mentioning that there were no substantial changes in the results when adjusting for radiographic TFOA, suggesting that our findings were not confused by radiographic TFOA.

We recognize several limitations in this study. Firstly, CT was used to define the PFOA. CT is not as sensitive as MRI in the detection of structural lesions. Nevertheless, all patients in this study were hospitalized for total knee arthroplasty or arthroscopic surgery, so our findings by CT were consistent with the results of further intraoperative observation, which suggested the robustness of our findings. Secondly, the patella alignment was measured by CT scans during knee extension. Therefore, the influence of the patella and femoral movement during knee flexion cannot be considered when measuring patella alignment. In the future, it is necessary to measure and analyze the PFJ alignment at different flexion angles. Thirdly, our study only analyzed the imaging findings, which need to be studied in combination with the patient’s symptoms in the future.

## Conclusions

In conclusion, our results identified that there was a U-shaped association between the patellar height and the severity of lateral PFOA. Moreover, patella lateral tilt, lateral displacement, and type III patella are also associated with the severity of lateral PFOA. This study is of clinical significance because identifying the role of PFJ malalignment and abnormal morphology in the severity of PFOA could provide early intervention strategies for patients who may increase the risk of developing PFOA.

## Data Availability

The detailed data and materials of this study are available from the corresponding author via e-mail on reasonable request.

## Abbreviations

PFOA: Patellofemoral osteoarthritis
PFJ: Patellofemoral joint
TTTG: Tibial tubercle–trochlear groove distance
TFOA: Tibiofemoral osteoarthritis
